# Medical Students' Experience of and Reaction to Stress: The Role of Depression and Anxiety

**DOI:** 10.1155/2014/737382

**Published:** 2014-01-29

**Authors:** Coumaravelou Saravanan, Ray Wilks

**Affiliations:** Division of Psychology (& Behavioural Sciences), International Medical University, No. 126, Jalan Jalil Perkasa 19, Bukit Jalil, 57000 Kuala Lumpur, Malaysia

## Abstract

*Background*. Medical school is recognized as a stressful environment that often has a negative effect on students' academic performance, physical health, and psychosocial well-being. Previous studies have not identified differences between depressed and nondepressed and anxious and nonanxious medical students' experiences of stress or their reactions to stressors. The present study aimed to identify the prevalence of depression and anxiety among a sample of 358 medical students attending a private university in Malaysia and to examine differences according to participants' gender, year of study, and stage of training (preclinical and clinical). Additionally, this study examined the extent to which stress predicts depression and anxiety, differences between depressed and nondepressed medical students' experiences of and reactions to stressors, and differences between anxious and nonanxious medical students' experiences of and reactions to stressors. *Methods*. The Student Life Stress Inventory was used to measure stress and reaction to stressors and the Depression, Anxiety, and Stress Scale was used to measure depression and anxiety. *Results*. The results showed that 44% (*n* = 158) of the students were anxious and 34.9% (*n* = 125) were depressed. More female students exhibited anxiety compared to male students. Stress is a predictor for depression and anxiety. A significant difference was found between depressed and nondepressed and anxious and nonanxious students' experience of stressors due to frustration, change, and their emotional reaction to stressors. *Conclusion*. Overall, depressed and anxious students were found to experience more stress and react differently to stressors compared to nondepressed and nonanxious students.

## 1. Introduction

The mental health of university students is an area of increasing worldwide concern as this population has been shown to be particularly prone to depression, anxiety, and stress due to factors that include academic pressures, obstacles to their goal achievement, environmental changes, and life challenges such as transition from school to university and the change in role from student to knowledgeable physician [1, 2].

Medical students have been found to experience higher levels of depression and anxiety compared to the general population and to their same age peers [3, 4]. Differences between levels of depression and anxiety have also been noted between medical students attending public and private medical schools. The prevalence of depression among medical students in public universities has been estimated to be 10.4% in Greece [5], 15.2% in USA [6], 21.7% in Malaysia [7], 24% in UK [8], 29.1% in India [9], and 43.8% in Pakistan [10]. The prevalence of depression among private medical students, however, has been estimated to be 19% in USA [11], 49.1% in India [12], and 60% in Pakistan [13].

The prevalence of anxiety among medical students attending public universities has been estimated to be 43.7% in Pakistan [14], 54.5% in Malaysia [15], 65.5% in Greece [5], and 69% in Beirut [16], while the prevalence of anxiety among students attending private medical colleges has been estimated to be 29.4% in Israel [17], 56% in India [18], and 60% in Pakistan [13].

### 1.1. Gender, Year of Study, and Stage of Training (Preclinical and Clinical)

Evidence for a relationship between medical students' gender, year of study, and stage of training (preclinical and clinical) and the development of depression and anxiety is equivocal. Some studies of medical students from public universities have reported that female students experience more depression, anxiety, and stress compared to male students [1, 19], while others have reported no gender difference in prevalence of depression [20]. Studies of medical students from private universities have identified that male students experience more depression and anxiety compared to female students [12, 18]. Regarding year of study, some studies of medical students from public universities suggest students in their first year of study exhibit higher levels of depression and anxiety compared to students in higher years of study [1, 21, 22]. A similar finding has been noted among students attending private universities where first and second year students exhibited higher levels of depression compared to those of students in higher years of study [12, 13]. However, Karadag and Nurcan identified no difference in depression levels according to students' year of study [23]. In a study conducted in a public university, it was identified that students undertaking clinical training exhibited higher depression levels compared to preclinical students [24]. Studies conducted in private universities found preclinical students exhibit more depression compared to students undertaking clinical training [12, 17].

Given the inconsistent findings regarding the relationship between levels of depression and anxiety and gender, year of study, and stage of training (preclinical or clinical) and the fact that studies have typically been undertaken in public universities in Malaysia and other countries, there is a need to further investigate these relationships and to investigate them among students in medical schools in private universities in Malaysia.

### 1.2. Stress as a Predictor of Depression and Anxiety

Stress has been found to correlate with depression and anxiety [2]. Previous studies have noted that various stressors, such as financial, workload, academic pressure, inadequate teacher and student relationships, parent and child relationships, family problems, peer relationships, physical illness, emotional problems, and worries about the future, contribute to poor mental health in some but not all medical students [15, 25, 26]. These studies, however, did not specifically explore the stressors faced by students suffering from either depression or anxiety. Past studies have identified that academic stress, relationships, distress, inability to enjoy normal activities, difficulties, facing situations, inability to overcome difficulties, and lack of concentration are the major stressors associated with depression but not anxiety among medical students [2, 7]. Untreated depression and anxiety are likely to be related to a student's suicidal behaviour, poor scholastic performance, and withdrawal from their medical course. Identifying the precipitating stressors for depression and anxiety may facilitate clinicians' and educational organisations' recognition of the important stressors faced by depressed and anxious students during their medical program.

The recent rapid increase in the number of medical schools in Malaysia from 21 (10 public and 11 private) in 2007 [21] to 31 (11 public and 20 private) in 2013 [27] makes Malaysia a fertile field for exploring a range of issues related to medical education. Among these are issues related to recruitment of appropriately qualified and experienced staff, physical infrastructure, quality of training, curriculum methods, future oversupply of medical practitioners, and, importantly, the mental health of students. Given the concern about international findings related to the mental health of medical students, there is an urgent need to assess these findings in the Malaysian context. While much of the international research into the mental health of medical students has been conducted in public medical schools, the rapid and disproportionate increase in the number of private medical schools in Malaysia provides an opportunity to conduct this type of research in a private medical school.

Therefore, among a sample of medical students attending a private medical university in Malaysia, the present study aimed to identify the

1. prevalence of depression and anxiety,
2. relationship between demographic variables (gender, year of study, and stage of training (clinical and nonclinical) and levels of depression and anxiety,
3. extent to which stressors and reactions to stressors predict depression and anxiety,
4. extent to which stressors (frustrations, conflicts, pressures, changes, and self-imposed) and reactions to stressors (physiological, emotional, behavioural, and cognitive appraisal) vary between students with depression and anxiety and students without depression and anxiety.

## 2. Methods

### 2.1. Participants

In this cross-sectional study, participants were 358 medical students who were attending a private university in Malaysia and who had completed at least six months of their medical degree (see Tables 2 and 3 for demographic details of the sample).

### 2.2. Measures

The questionnaire package used in this study consisted of three components: a sociodemographic questionnaire that required each student to provide their age, gender, and year of study; a measure of student life stress (The Student Life Stress Inventory); and a measure of depression anxiety (the Depression, Anxiety, and Stress Scale).

#### 2.2.1. The Students Life Stress Inventory (SSI)

The SSI is a 51-item inventory designed to assess students' perceived academic stress and reactions to stress. Responses are provided using a Likert scale that ranges from 1 = never true to 5 = always true. The inventory assesses five categories of academic stressors (frustrations, conflicts, pressures, changes, and self-imposed) and four categories describing reactions to stressors (physiological, emotional, behavioural, and cognitive appraisal). Validity and reliability of the instrument have been found to be adequate and reported internal consistency estimates ranging from 0.69 to 0.82 across the nine categories [28].

#### 2.2.2. The Depression, Anxiety, and Stress Scale (DASS-21)

The DASS-21 was used in the present study; however, only the depression and anxiety scores were considered because stress was more comprehensively measured by the SSI. The DASS-21 is reported to have very good Cronbach's alpha values for depression and anxiety (.84 and .74, resp.) [29].

### 2.3. Procedure

Following the granting of ethical approval from the university to conduct the study, medical students in years 1–5 were contacted after the conclusion of one of their lectures and were invited to participate in the study. Students who agreed in writing to participate were each given a questionnaire package to complete and return to the researcher before leaving the lecture room.

## 3. Results

Data were analyzed using PASW version 18.0 (SPSS Inc., 2009) [30].

### 3.1. Prevalence of Depression and Anxiety

The prevalence level of depression and anxiety among the sample is shown in Table 1. Some level of depression was reported by 34.9% of the sample while some level of anxiety was reported by 44% of the sample.

### 3.2. Relationship between Gender, Year of Study, and Stage of Training (Clinical or Nonclinical) with the Existence or Nonexistence of Depression

Table 2 indicates no significant relationship between gender, year of study, or stage of training (preclinical or clinical) with the existence or nonexistence of depression.

### 3.3. Relationship between Gender, Year of Study, and Stage of Training (Clinical and Nonclinical) with the Existence or Nonexistence of Anxiety

Table 3 shows a significant relationship between gender and anxiety (*χ* ^2^ = 10.3 (1), *P* = 0.01) and a nonsignificant relationship between year of study or stage of training (preclinical or clinical) and anxiety.

### 3.4. Stress as a Predictor of Depression and Anxiety

For the regression model, this study used total stress score as a predictor variable and total anxiety and depression score as criterion variables.  Table 4 indicates that stress is a significant predictor of depression (*β* = 2.11, *t*(355) = 4.06, *P* < 0.001) and anxiety (*β* = .201, *t*(355) =  3.87, *P* < 0.001) and predicts 44% (*R* ^2^ = .44, *F*(1,355) = 16.51, *P* < 0.001) of depression and 40% (*R* ^2^ = .40, *F*(1,355) = 14.97, *P* < 0.001) of anxiety.

### 3.5. Difference between Anxious and Nonanxious Students' Stressors and Reaction to Stressors

Table 5 shows that there is a significant difference between anxious and nonanxious students' total stressors score (*t*(356) = −2.714, *P* = 0.007, *d* = 0.28) and total reaction to stressors score (*t*(356) = −2.80, *P* = 0.005, *d* = 0.38). Significant differences are shown between anxious and nonanxious students' responses to three of the five stressor subscales: frustration (*t*(356) = −2.406, *P* = 0.018, *d* = 0.25), pressure (*t*(356) = −2.402, *P* = 0.017, *d* = 0.25), and changes (*t*(356) = −2.230, *P* = 0.026, *d* = −.24). Significant differences are shown between anxious and nonanxious students' physiological (*t*(356) = −2.895, *P* = 0.004, *d* = 0.30) and emotional (*t*(356) = −2.285, *P* = 0.023, *d* = 0.24) response to stressors. The total mean values of stressors (*M* = 71.59) and reaction to stressors (*M* = 70.76) are higher for anxious students compared to nonanxious students' mean value of stressors (*M* = 67.99) and reaction to stressors (*M* = 65.72). The Cohen effect size value (*d*) suggested a smaller to medium significance between anxious and nonanxious students' stressors and reactions to stressors.

### 3.6. Difference between Depressed and Nondepressed Students' Stressors and Reaction to Stressors

Table 6 shows that there is a significant difference between depressed and nondepressed students total stressors scores (*t*(356) = −2.12, *P* = 0.034, *d* = 0.23); however, no significant difference exists between depressed and nondepressed students' total reaction to stressors score (*t*(356) = −1.64, *P* = 0.101). A significant difference exists between depressed and nondepressed student's responses to two of the stressor subscales: frustration (*t*(356) = −2.056, *P* = 0.044, *d* = 0.22) and changes (*t*(356) = −2.57, *P* = 0.010, *d* = 0.29). A significant difference exists between depressed and nondepressed students' emotional reaction to stressors (*t*(356) = −2.03, *P* = 0.043, *d* = 0.22) but not to their physiological, behavioural, or cognitive reaction to stressors. The total mean value of depressed students' stressors level is higher (*M* = 71.52) compared to the total mean value (*M* = 68.54) of nondepressed students. The Cohen effect size value (*d*) suggested a smaller to medium significance between depressed and nondepressed students' stressors and reaction to stressors.

## 4. Discussion

International research has identified concern about the high levels of depression and anxiety among medical students attending either public or private medical schools. While some research indicates similarly high levels among students attending Malaysian public medical schools, little is known about the situation in Malaysian private medical schools. Given the recent considerable increase in the number of private medical schools in Malaysia, the current research investigated the prevalence of depression and anxiety in a Malaysian private medical school and examined relationships between gender, year of study, and depression and anxiety. In addition, this research has investigated differences between students with and without depression and anxiety regarding their stressors and reactions to stressors. Finally, stress as a predictor of depression and anxiety was examined.

*Prevalence of Depression and Anxiety.* The prevalence of depression among the sample of medical students in the present study was 34.9%, a prevalence that is higher than was found in a study of medical students attending an American private university (19%) [11] but lower than was found in studies of medical students attending private universities in India (49.1%) [12] and Pakistan (60%) [13]. Compared with studies of medical students attending public universities, the prevalence of students with depression in the present study is higher than was found among medical students attending public universities in Greece (10.4%) [5], USA (15.2%) [6], Malaysia (21.7%) [7], UK (24%) [8], and India [9]. The results of the present study confirm the strong trend in the literature that medical students attending private medical schools exhibit more depression than students attending public medical schools. This trend may be due to additional pressures placed on students due to high expectations from parents who have made a substantial financial investment in their child's private medical education. Expectations from faculty in private medical schools may be greater than expectations from faculty in public medical schools. This difference may be due to a perception by faculty in private medical schools that, because students (parents) are paying higher fees, more is expected from the faculty. The higher prevalence of depression in students attending private medical schools may be also due to particular characteristics of students who choose to study at a private institution. A clearer understanding of the high prevalence of depression among medical students attending private medical schools, however, is the focus for future research and is beyond the scope of the present investigation.

The prevalence of anxiety among students in the present study was 44%, a prevalence rate that lower than is found in private medical colleges in a number of countries, for example, India (56%) [18] and Pakistan (60%) [13], but higher than is found in others, for example, Israel (29.4%) [17]. Compared to the prevalence of anxiety among students attending public medical schools, the current prevalence rate is lower than those found in many countries, for example, Greece (65.5%) [5], Malaysia (54.5%) [15], and Beirut (69%) [16]. The reasons why the prevalence rate of anxiety in the present sample of students is generally lower than those reported in other studies are unclear and identify a need for further research in this area.

*Relationships between Gender, Year of Study, and Depression and Anxiety.* The results of this study show no significant relationship between gender and depression, a result also reported by a previous study [31]. The trend in the present study that more female students tended to experience depression than male students is similar to that found by a previous study [32].

A significant relationship, however, was found in the present study between gender and anxiety where more females than males experienced anxiety. This result is in contrast to those reported for students in some other private medical schools [12, 13] but consistent with those reported for students in some public universities [19, 33]. The trend that females experience more anxiety than males may suggest that female medical students are more competitive, tend to be more concerned about working hard to secure higher marks in exams, are more concerned about their performance, exaggerate their sadness, and tend to engage in less exercise [13, 34]. More research is clearly needed before these suggestions can be confirmed or denied.

Regarding relationships between year of study stage of training (clinical or nonclinical), and depression and anxiety, results from the present study indicate no statistically significant relationships; however, a trend can be seen that suggests depression and anxiety increases as students progress through their medical training. The only exception to this trend occurs in the first year where the prevalence of depression is greater than in any other year. This exception may be due to a number of unique stressors facing first year students that relate to the transition from secondary school to university, homesickness, unfamiliarity with academic procedures and demands, time management, the process of making new friends, and increased expectations from family and faculty. The increase in the prevalence of both depression and anxiety as students progress through their programme is generally noted in the literature along with the identification of associated physical and mental health implications of anxiety and depression [3, 12, 19, 35, 36]. These findings have important implications for medical schools to ensure their programmes do not place excessive pressures on their students and to provide students with techniques to manage their own levels of anxiety and depression. The provision of self-help programmes at the commencement of and throughout medical training should be an integral component of all medical training programmes along with the availability of counselling resources at each university. During students' clinical training, it is important that medical schools not only provide their students with adequate clinical knowledge and training with simulated patients but also provide opportunities for their students to identify, discuss, and manage their own personal issues.

*Stressors and Reactions to Stressors.* Significant differences were found in the present study between the types of stressors experienced by anxious and nonanxious students and depressed and nondepressed students. Anxious students experienced significantly more frustrations (related to failure to accomplish the work, daily hassles, and delays in reaching goals), pressures (due to deadlines, overwork, and conflicts in interpersonal relations), and changes (rapid and too many occurring at the same time) than did nonanxious students. Their reactions to stressors were also significantly different. Anxious students reacted more physiologically (stuttering, trembling, and rapid movements) and emotionally (fear, anxiety, worry, grief, sad mood, and guilt) than did nonanxious students. These results are consistent with previous findings that indicate medical students who are anxious experience major stressors such as lack of concentration, heavy workload, many assignments, too many test activities, frequent strain, and inability to make decisions and inability to answer for lecturers [2, 19, 37].

In this study depressed students experienced significantly more stressors due to frustration and too many changes occurring at the same time than nondepressed students. In addition, depressed students exhibit more emotional reaction of stress than nondepressed students. This study did not find significant differences on conflict, pressure, and self-imposed stressors and physiological, behavioural, and cognitive reactions to stressors among depressed medical students. Previous research has, however, suggested that medical students who are depressed experience more affective, cognitive, and somatic problems [24]. The results of this study consistent with previous study that depressed students were experiencing frustration due to too many assignments [19] and being unable to concentrate constantly under strain and the feeling of worthless [2].

Private universities expect their students to be more competitive and, to achieve their targets, students have to work hard. Private university students exhibit more psychological problems and stress due to the new study environment, financial indebtedness, homesickness, and greater degree of workload with obligations to succeed. Due to the workload, they often experience a lack of sleep, leisure activities, and exercise. In addition, parents may pressurise their children to work hard as the tuition fees are higher at private universities [38] and high parental expectations also cause undue stress [37]. These stressors gradually decrease when students progress in their medical programme but some students may not be able to overcome these stressors which may lead to poor academic performance, substance abuse, and mental illness [12, 13, 34].

*Stress as a Predictor of Depression and Anxiety.* Stressors were the significant predictor of anxiety and depression found in this study. Previous studies also found that examination criteria, too many test activities, lack of leisure time, work pressure, and overworkload are the major stressors for the development of anxiety and depression among medical students [19, 37].

Universities and students counsellors are to focus on stressors such as frustration, pressure, and changes as these stressors are existing more among depressed students compared to nondepressed students. Identifying these stressors at the early stage in the medical programme will prevent students from suffering from depression and anxiety. Conducting stress management workshop, providing awareness about stressors, and availability of mental health services during orientation day will be beneficial for the students to have insight about stress, depression, and anxiety. Scholastic related workload in the field of medical education is unavoidable and universities have to teach time management and study management skills for the students to manage their work pressure and frustration.

This study has a few limitations. One of the limitations is that this study did not focus on other causes for depression and anxiety such as personality, demographic information, and family status. Second limitation is that the findings of this study may not be generalized as the results are based upon one private university in Malaysia. Future study would focus on what coping skills used by students with depression and anxiety and without depression and anxiety are. In addition future study would be conducted on providing opportunity to participate in stress management and study skill management workshop or individual training in the first semester of the medical program prevent the anxiety and depression or not.

This study concluded that female students experienced more anxiety compared to male students. Stressors were the predictor of depression and anxiety. Universities have to consider the stressors due to frustration, pressure, and conflict and their physiological and emotional reactions to stressors.

## Figures and Tables

**Table 1:** Prevalence of levels of depression and anxiety.

		*n* (%)
Level of depression	None	233 (65.1)
	Mild	73 (20.4)
	Moderate	46 (12.8)
	Severe	4 (1.1)
	Extreme	2 (.6)

Level of anxiety	None	200 (55.9)
	Mild	65 (18.2)
	Moderate	71 (19.8)
	Severe	21 (5.9)
	Extreme	1 (.3)

**Table 2:** Relationship between gender, year of study, and stage of training (clinical or nonclinical) with the existence or nonexistence of depression.

	All participants (*n* = 358)	Nondepression	Depression	*χ* ^2^ (df)	*P* value
	*n* (%)	*n* (%)	*n* (%)		
Gender					
Male	177 (49.4)	116 (65.5)	61 (34.5)	.032 (1)	.852
Female	181 (50.6)	117 (64.6)	64 (35.4)		
Year of study					
1st	89 (24.9)	50 (56.2)	39 (43.8)	5.54 (4)	.236
2nd	93 (26.0)	63 (67.7)	30 (32.3)		
3rd	71 (19.8)	52 (73.2)	19 (26.8)		
4th	67 (18.7)	44 (65.7)	23 (34.3)		
5th	38 (10.6)	24 (63.2)	14 (36.8)		
Stage of training					
Preclinical	261 (73.3)	170 (65.1)	91 (34.9)	.026 (1)	.872
Clinical	95 (26.7)	61 (64.2)	34 (35.8)		

**Table 3:** Relationship between gender, year of study, and stage of training (clinical and non-clinical) with existence or nonexistence of anxiety.

	All participants (*n* = 358)	Nonanxiety	Anxiety	*χ* ^2^ (df)	*P* value
	*n* (%)	*n* (%)	*n* (%)		
Gender					
Male	177 (49.4)	114 (64.4)	63 (35.6)	10.3 (1)	0.001*
Female	181 (50.6)	86 (47.5)	95 (52.5)		
Year of study					
1st	89 (24.9)	46 (23.0)	43 (27.2)	2.19 (4)	.700
2nd	93 (26.0)	56 (60.2)	37 (39.8)		
3rd	71 (19.8)	42 (59.2)	29 (40.8)		
4th	67 (18.7)	37 (55.2)	30 (44.8)		
5th	38 (10.6)	19 (50.0)	19 (50.0)		
Stage of training					
Preclinical	261 (73.3)	144 (55.2)	117 (44.8)	.079 (1)	.779
Clinical	95 (26.7)	54 (56.8)	41 (43.2)		

*P value less than 0.05 was defined as significant.

**Table 4:** Stress as a predictor of anxiety.

	*B*	Std. error	*β*
Anxiety	2.491	1.120	
Total stress score	.025	.006	.201**

Depression	2.781	1.197	
Total stress score	.028	.007	2.11**

Model 1 (anxiety) = Δ*R* ^2^ = .040; **P < 0.01; model 2 (depression); Δ*R* ^2^ = .044; **P < 0.001.

**Table 5:** Difference between anxious and non-anxious students' stressors and reaction to stressors.

Subscales	Nonanxious	Anxious	*t* (356)	*P* value
	*M* (Sd)	*M* (Sd)		
Stressors				
Frustration	17.98 (5.06)	19.21 (4.61)	−2.406	0.018*
Conflict	8.46 (2.32)	8.75 (2.23)	−1.204	0.229
Pressure	12.89 (3.28)	13.72 (3.20)	−2.402	0.017*
Changes	7.99 (2.88)	8.64 (2.52)	−2.230	0.026*
Self-imposed	20.57 (4.19)	21.22 (4.36)	−1.437	0.152
Total stressors score	**67.99** (**12.94**)	**71.59** (**12.09**)	−**2.714**	0.007*
Reaction to stressors				
Physiological	30.96 (11.38)	34.19 (9.74)	−2.895	0.004*
Emotional	11.89 (3.88)	12.75 (3.25)	−2.285	0.023*
Behavioural	16.11 (5.11)	16.79 (5.49)	−1.179	0.239
Cognitive appraisal	6.78 (1.97)	7.04 (1.86)	−1.290	0.198
Total reaction to stressors score	**65.72** (**17.90**)	**70.76** (**5.50**)	−**2.807**	0.005*

*P value less than 0.05 was defined as significant.

**Table 6:** Difference between depressed and nondepressed students stressors and reaction to stressors.

Subscales	Nondepression	Depression	*t* (356)	*P* value
	*M* (Sd)	*M* (Sd)		
Stressors				
Frustration	18.14 (4.97)	19.23 (4.70)	−2.056	0.044*
Conflict	8.57 (2.32)	8.62 (2.23)	−.212	0.832
Pressure	13.05 (3.41)	13.63 (2.95)	−1.688	0.92
Changes	8.00 (2.83)	8.78 (2.51)	−2.57	0.010*
Self-imposed	20.64 (4.28)	21.25 (4.26)	−1.276	0.203
Total stressors score	**68.54** (**12.70**)	**71.52** (**12.48**)	−**2.12**	0.034*
Reaction to stressors				
Physiological	31.73 (11.19)	33.59 (9.95)	−1.611	0.108
Emotional	11.98 (3.60)	12.80 (3.65)	−2.037	0.043*
Behavioural	16.19 (5.46)	16.18 (5.48)	−1.013	0.312
Cognitive appraisal	6.97 (2.04)	6.74 (1.70)	1.177	0.240
Total reaction to stressors score	**66.87** (**17.32**)	**69.93** (**16.39**)	−**1.64**	**0.101**

*P value less than 0.05 was defined as significant.
